# Heterogenous Tau Pathology in the Hippocampus of Progressive Supranuclear Palsy

**DOI:** 10.1002/alz.092937

**Published:** 2025-01-03

**Authors:** Merci N Best, Jonathan Levy, Amber Spencer, Lia Min, Einar Olafasson, Emile Pinarbasi, Jonny Sexton, Paul M Jenkins, Vernon B Carruthers, Henry L Paulson

**Affiliations:** ^1^ University of Michigan, Ann Arbor, MI USA; ^2^ Michigan Alzheimer’s Disease Research Center, Ann Arbor, MI USA

## Abstract

**Background:**

Globose neurofibrillary tangles (NFTs) are found in subcortical areas of post‐mortem brain from individuals with the second most common primary tauopathy, progressive supranuclear palsy (PSP). The degree of cognitive impairment in secondary tauopathies such as Alzheimer’s disease (AD) correlates with the presence of NFTs, which originally appear in the entorhinal cortex before spreading throughout the hippocampus. In contrast, the degree of hippocampal tau pathology in PSP is thought to be limited, consistent with the view that cognitive impairment in PSP is predominantly subcortical. To better define the extent and variability of tau pathology in subregions of the hippocampus (dentate gyrus, CA4, CA3, CA2, CA1, subiculum, and parahippocampal gyrus), we examined PSP brains available through the Michigan Brain Bank.

**Method:**

We identified 33 brains collected since 2011 where the primary neuropathology was PSP. For further analysis, we selected 15 brains (10 male), 14 of which lacked beta amyloid pathology. In 12 cases, clinical and neuropathological diagnoses of PSP were concordant (three other cases had been clinically identified as corticobasal degeneration or “other cognitive disorder”). Immunofluorescence microscopy and western blot analysis were performed with an antibody specific for phosphorylated tau, Tau_pS202/pT205_. A semi‐quantitative scale was used to determine the degree of NFT pathology (0 = negative, 1 = sparse, 2 = intermediate, and 3 = robust) in hippocampal regions of PSP.

**Result:**

Immunofluorescence demonstrated wide variability in the degree and range of tau pathology within the hippocampus. Five of 15 PSP brains demonstrated robust NFT pathology in at least one hippocampal subregion. Within the hippocampus, the CA1 and subiculum most commonly displayed denser NFT pathology whereas the dentate gyrus typically was spared. Western blot analysis likewise demonstrated marked heterogeneity in Tau_pS202/pT205_ signal, ranging from 0 to 161 arbitrary units.

**Conclusion:**

These findings support the view that hippocampal NFT pathology is not uncommon in PSP and may contribute to cognitive impairment in a subset of patients. Furthermore, the high variability in hippocampal NFTs nominates this region in PSP as a compelling brain area in which to assess correlations between tau accumulation and perturbations in neuronal cytoarchitecture.

